# Health in West Africa

**Published:** 1901-09-14

**Authors:** 


### HEALTH IN WEST AFRICA.

After describing the work which is now going on in the various towns on the West Coast of Africa for the abolition of the mosquito by the removal of their breeding beds, Major Donald Ross, of the Liverpool School of Tropical Medicine, makes some useful remarks as to the reputed unhealthiness of this part of the world. He says that the unhealthiness of the Coast has been much exaggerated. True, there is a considerable amount of malaria among Europeans, but everywhere we meet men who have lived for years on the Coast in a good state of health. It is the young, feckless, improvident, and sometimes intemperate newcomer who generally falls a victim to disease. The sober sensible man can usually rely upon escaping, and it may be said with confidence that in nine cases out of ten if a man contracts malarial infection it is his own fault. The scrupulous use of a mosquito net, attention to domestic cleanliness, exercise, temperance, and an occasional strong dose of quinine, are the things principally required. If men, he says, despise every dictate of science, as well as of common sense, what can they expect? Intemperance is a potent ally of malaria, not only directly but also indirectly, for some of the worst malarial infections are undoubtedly acquired in the houses of native women. But even if a man does acquire a malarial infection a three months' course of quinine ought to rid him of it. As a rule, quinine is taken only when there is fever, and is stopped just when it begins to produce a good effect. Dr. Ross' account is thus very satisfactory, and shows well what advances are being made in the control of one of the most deadly of diseases. At the same time it must be remembered that what is now being done is practically confined to towns, and that those whose duties take them up to the country and compel them to sleep in ill-built houses in the neighbourhood of natives villages still run great risks, and that it is far too early to make any statement which should lead to any mitigation of the general ill repute which hangs over the whole of this district, or to any lessening of the high rewards, in the way of pay and leave, which have hitherto been found necessary to induce men to take up their residence in this unattractive part of the world.

